# A data-centric perspective on designing AI foundation models for healthcare

**DOI:** 10.3389/fdgth.2026.1738523

**Published:** 2026-04-20

**Authors:** Baraa Al Jorf, Bartlomiej Piechowski-Jozwiak, Farah E. Shamout

**Affiliations:** 1 Engineering Division, New York University Abu Dhabi (NYUAD), Abu Dhabi, United Arab Emirates; 2 Department of Computer Science and Engineering, NYU Tandon School of Engineering, New York, NY, United States; 3 Department of Neurology, King's College Hospital, London, United Kingdom

**Keywords:** artificial intelligence, clinical AI, foundation models, health informatics, healthcare, machine learning, multimodal data

## 1 Introduction

Healthcare data comprises diverse data modalities, such as electronic health records, medical images, radiology reports, and clinical notes [1]. When effectively analyzed and integrated using machine learning, multimodal data holds immense potential to enable precision medicine [2, 3]. Foundation models, such as large language models built using deep neural networks and large-scale datasets, have driven a paradigm shift in AI. In healthcare, foundation models show promise for advancing generalist medical AI with their advanced capabilities [4]. Specifically, AI foundation models are uniquely suited to handle multimodal data and have already demonstrated substantial improvements in performance in various downstream clinical tasks [5, 6]. Integrating these models into clinical workflows can help streamline labor-intensive tasks and reduce administrative and cognitive burdens on healthcare providers, allowing them to prioritize patient care [7, 8].

Despite AI’s promise of improving clinical workflows, healthcare professionals remain concerned about the black-box nature of AI systems, which limits clinical acceptance and confidence [9–12]. Design choices for developing AI models generally vary significantly due to the complex landscape of clinical data and practitioner requirements. This in turn hinders real-world deployment, due to lack of generalizability, considering the significant heterogeneity across institutions [5, 13], and lack of transparency, especially pertaining to data quality [14]. Additionally, AI foundation models rely heavily on the availability of large-scale data, which are notoriously difficult to collect and annotate in healthcare settings [14]. Hence, there is an imperative need for standardization and increased transparency, to ensure responsible deployment of AI foundation models in healthcare.

Furthermore, recent literature reinforces a clear need for a data-centric perspective in the development of foundation models. Establishing regulatory frameworks for these models remains challenging because of their broad versatility and the difficulty of anticipating their downstream clinical impact when adapted across tasks and institutions [15]. At the same time, emerging analyses argue that data represents the core challenge for foundation models, pointing to unresolved issues related to ethics, diversity, cost, and heterogeneity of large-scale datasets [5]. In addition, inconsistencies in how clinical data is managed across different studies complicate reproducibility. For example, electronic health record data are treated as a single unimodal source in some studies [6, 16], whereas others decompose them into multiple components that require distinct preprocessing pipelines [17, 18]. The role of demographic variables is similarly debated, with some studies reporting performance or fairness benefits and others raising concerns about bias amplification [19, 20]. Such observations underscore the absence of a coherent data-level framework.

Consequently, we aim to identify key principles to guide the design, development and evaluation of AI foundation models in healthcare. Although general guidelines for AI design and human-AI interaction exist, they tend to be broad or insufficiently tailored to the specific needs of healthcare applications [21, 22]. Healthcare-oriented frameworks primarily address technical integration and operationalization into electronic health records, but lack detailed guidance on model design considerations [23]. These limitations underscore the need for targeted, expert-informed guidance that explicitly addresses the nuanced data-centric challenges unique to healthcare applications. To fill this gap, we articulate a data-centric perspective on designing, developing, and evaluating AI foundation models for healthcare. Informed by our reading of the literature and ongoing dialogues with clinicians, we distill 28 principles specifically aimed at guiding the development of clinically relevant AI foundation models. Our main contribution is the consolidation of existing best practices and data considerations within a unified framework. The target audience includes AI researchers and clinicians interested in the development, evaluation and adoption of such models. Adopting this framework could ensure that future models are not only technically robust, but also closely aligned with practical real-world challenges.

## 2 Data-centric principles for AI foundation models in healthcare

Several studies have established guidelines for specific aspects of AI development, focusing on areas such as human-AI interaction [21], generative AI design [22], or post-hoc explainability (XAI) [24]. Other foundational standards like the FAIR principles (Findable, Accessible, Interoperable, Reusable) govern data management [25], while frameworks like DEPLOYR guide the technical operationalization of models into electronic medical records [23]. While these efforts are vital, they largely overlook the a priori data-facing decisions required to build robust clinical foundation models. To bridge this gap, we propose a data-centric perspective specifically tailored for designing foundation models in healthcare. This perspective tackles specific challenges such as data heterogeneity, sparsity, and low effective sample size, which could lead to significant downstream limitations.

In particular, clinical data is rarely homogeneous or fully complete. Hence, heterogeneity remains a major issue for integrating routine clinical data across settings, due to fragmented, incomplete or inconsistent data sources [26]. Similarly, data quality and bias have been identified as limiting factors when it comes to clinical integration [27]. Our proposal for a data-centric framework would directly prioritize these issues by encouraging structured harmonization of modalities and terminologies, explicit quantification and handling of missingness and other structural limitations, and bias-aware data-curation to ensure models are representative of the target patient population during model training and development.

Our proposed principles are presented in Table 1, divided across three stages: data curation and preprocessing, model design and development, and evaluation. Each stage encompasses specific design principles to be considered for developing an AI foundation model that could potentially be deployed in clinical settings. We first identified a preliminary set of principles based on a literature review of 63 existing studies focused on multimodal foundation models in healthcare. We structured the guidelines based on the general end-to-end machine learning pipeline, from data curation up to model evaluation. The principles were then formalized via an iterative process with a group of ten clinical experts and ten AI practitioners. We invited practitioners who were actively engaged in AI research and development to a roundtable discussion as part of a clinical AI bootcamp. Each roundtable consisted of 5-6 participants and 1-2 discussion leads. The discussion leads were provided with a guiding document and questions related to handling and processing multimodal data, and were responsible for collating the group’s feedback. Clinicians provided insights into the nuance of medical records and workflow constraints, while AI practitioners assessed the technical feasibility of the proposed data curation strategies from the specific perspective of multimodal foundation models. After refining the principles, we conducted focus group sessions with the AI experts who evaluated the draft principles against real-world case studies, identifying practical bottlenecks in data access and integration. This feedback loop allowed us to refine the principles to a recommended set of actionable guidelines that bridge the gap between modeling requirements and the reality of clinical practice.

**Table 1:** Our table of data-centric guidelines for the development of trustworthy FMs in clinical settings.

Stage	Design principle	Guidelines
Data curation & preprocessing	1. Consistent definition of a data sample	1A. Identify relevant data modalities a priori and justify inclusion based on clinical value for the defined target population.
		1B. Define the number and type of data modalities that represent a single sample.
		1C. Prioritize cost-effective and routine data modalities, unless use of more expensive modalities is clinically justified.
		1D. Specify at which level multiple modalities are being grouped to form a sample, such as on a patient- or admission-level.
		1E. Develop data synchronization, or pairing, strategies if data modalities are collected at different time points.
		1F. Describe any differing assumptions underlying training and test samples.
	2. Accounting for data sparsity	2A. Identify and quantify the extent of missing data for each modality.
		2B. Design and justify an inclusion and exclusion criteria for dealing with missing data.
		2C. Specify how data missingness is dealt with during training and inference, and justify any assumptions or differences.
	3. Large-scale data integration	3A. Aggregate datasets collected in different settings to capture variability across clinical scenarios.
		3B. Specify dataset size for unimodal and multimodal samples.
		3C. Link data samples with ground-truth labels for the evaluation tasks.
	4. Acknowledgment of data bias	4A. Assess potential sources of bias in the data.
		4B. Ensure the datasets adequately represent the defined target clinical population or explicitly note deviations.
		4C. Justify the inclusion of specific attributes such as socioeconomic status or demographic information.
Model design & development	5. Representation learning	5A. Determine the learning objectives for representation learning.
		5B. Specify model capabilities. i.e perception, reasoning, or generation.
		5C. Designate the architecture of neural network encoder for each data modality.
		5D. Determine cross-modal learning strategy in case of multiple modalities, such as fusion, alignment, or translation.
		5E. Incorporate design features that enhance model explainability wherever feasible.
	6. Computational requirements	6A. Specify number of trainable and non trainable parameters of the model.
		6B. Estimate memory size of a single unimodal or multimodal sample and associated batch size during training.
		6C. State hardware resources used and report associated training and inference time.
Evaluation	7. Empirical assessment	7A. Specify downstream evaluation tasks.
		7B. Select and justify choice of evaluation performance metrics.
		7C. Evaluate performance across different patient subgroups to assess for potential biases.
		7D. Implement strategies to address bias during training and evaluation.
		7E. Conduct ablation study to assess impact of different design choices, such as multimodality.

### 2.1 Data curation and preprocessing

Recent work related to AI foundation models in healthcare focus on learning using diverse sources of information, with varying temporal and structural constraints. Hence, in the first stage, the main design principles are related to ensuring a consistent definition of a data sample, accounting for data sparsity, integrating large-scale data, and dealing with data bias. To ensure clinical value, a clear articulation of the intended patient population for whom the AI model is being developed is advised [28]. This should include specific inclusion and exclusion criteria based on clinical characteristics, e.g., age, specific diagnoses, disease severity, comorbidities, prior treatments, so that the model has external validity similar to clinical trials [29]. Additionally, determining whether the data should be aggregated at the patient or admission level, depending on the expected prediction tasks, and specifying how the different modalities align in time are essential steps to capture clinically meaningful events [8, 30]. These steps help ensure that integrated representations reliably reflect the underlying patient states, rather than arbitrary data concatenations [31]. The principles also guide developers to predefine and justify inclusion and exclusion criteria, identify extent of data missingness, and assess potential sources of bias to ensure equity and access [19, 32]. Such practices are aligned with recommendations to ensure high clinical utility of AI models and regulatory approval [33, 34].

### 2.2 Model design and development

Designing AI foundation models for healthcare requires careful choices that ensure adequate representation learning and downstream operational feasibility [2, 30]. This stage emphasizes defining clear learning objectives and model capabilities, designating the neural network architecture and cross-modal learning strategy [6, 35], and incorporating design features that ensure explainability [36, 37]. These steps directly impact the model’s ability to learn useful representations to support the high-stakes nature of clinical decision-making. Furthermore, in this stage, we highlight the need for specificity regarding the model’s computational footprint, such as model size and memory requirements. This enables the alignment of performance goals with real-world hardware constraints and workflow patterns, ensuring that even advanced architectures can be smoothly integrated into existing clinical workflows.

### 2.3 Evaluation

Comprehensive and extensive evaluation is crucial for establishing the utility and generalizability of AI foundation models. While this primarily entails careful selection and justification of the downstream tasks and evaluation metrics, the guidelines also encourage developers to conduct a subgroup analyses to uncover performance disparities across patient demographics for fairness purposes [23, 38]. Merely reporting population-level performance metrics, like area under the curve, may obscure biases that affect clinical equity [39]. For example, certain problems may require metrics that capture rare event detection or calibration quality, factors that matter significantly more in a clinical context than they do in a controlled research environment [5]. Adopting principles of rigorous evaluation is critical for transitioning AI foundation models from experimental settings into safe and effective real-world deployment.

### 2.4 Illustrative example: multimodal foundation model for sepsis

To demonstrate the practical utility of the proposed guidelines for developing multimodal foundation models, we consider the example of designing a model to predict the risk of sepsis during a hospital stay in a retrospective study. The example provided is strictly a hypothetical design scenario. Our framework imposes a structured design process across three stages. First, because sepsis prediction is an inherently multimodal problem where patient decline manifests across various clinical dimensions [40], we identify two primary data modalities (Principle 1). We select chest X-ray imaging, which supports the diagnosis of pneumonia, the most common cause for sepsis, alongside clinical data extracted from the electronic health record (EHR). These EHR records are highly relevant, as they capture the systemic responses critical to identifying sepsis, including vital signs and laboratory test results such as heart rate, temperature, and white blood cell count. Second, because our goal is to enable real-time prediction, we define each data sample as a 12 h window of physiological observations paired with the most recent chest X-ray image. This 12 h window is specifically chosen because it provides a sufficient timeframe to capture acute physiological trends and rapid deterioration, while remaining short enough to allow for timely clinical intervention. We assume no differences between training and test distributions and require the presence of both modalities for each sample during training and inference. Consequently, we exclude samples without an associated chest X-ray, recognizing that all patients have baseline clinical data (Principle 2). Given that foundation models demand substantial scale, we further assume that heterogeneous datasets from multiple clinical sites will be integrated to achieve sufficient sample size (Principle 3), with explicit documentation of sample counts at both the unimodal and multimodal levels, as well as systematic linkage of each sample to sepsis outcomes based on diagnosis codes, procedures, and medication records. To support fairness considerations, we additionally document the demographic composition of the development cohort, including age, sex, and other characteristics that may signal potential biases (Principle 4).

During the modeling phase, we propose the use of pre-trained modality-specific encoders trained with contrastive objectives. For example, a vision transformer for chest X-ray interpretation and an LSTM-based encoder for vital signs and labs, reflecting the core perceptual and temporal reasoning capabilities required for the task (Principle 5). The model’s final prediction relies on fusing the latent representations of the two modalities while respecting the computational and memory constraints of standard hospital hardware, including specifications related to GPU availability, CPU capacity, and the number of trainable and non-trainable parameters across stages, as well as estimated memory usage during training and inference (Principle 6). Finally, the validation phase focuses on evaluating performance using conventional metrics for sepsis prediction, such as accuracy, AUROC, and AUPRC, and moving beyond aggregate metrics to conduct granular subgroup analyses across patient demographics, performing post-hoc calibration when miscalibration is detected in specific subpopulations, and carrying out ablation studies to quantify performance degradation in the presence of missing modalities (Principle 7). These steps demonstrate how the proposed data-centric principles could guide the development and evaluation of robust multimodal foundation models prior to pursuing silent evaluation, prospective validation, pilot studies, and deployment.

## 3 Conclusion

We propose data-centric principles to support the development of AI foundation models that are accurate and efficient. By adhering to these principles, we anticipate improved transparency and reproducibility. This, in the long term, would accelerate the integration of AI technologies into healthcare systems, enhance decision-making processes, and, most importantly, improve patient outcomes. As we move forward, it is crucial for the AI and medical communities to collectively prioritize common considerations to unlock the full transformative potential of AI in healthcare.

The collaborative nature of our approach highlights the necessity of continuous dialogue between clinicians, engineers, and researchers. By fostering interdisciplinary collaboration, we can ensure that AI tools are not only technically sound but also address the ethical, operational, and practical needs of healthcare providers and patients. One key limitation of our framework is that it is purely conceptual, and its practical value will need to be assessed through applications in healthcare settings. Future work should focus on refining these guidelines through real-world case studies, where their utility can be further validated across diverse clinical environments and use cases. Additionally, exploring strategies to operationalize these principles in resource-constrained settings, such as low- and middle-income countries, remains an essential avenue for making AI foundation model advancements more equitable and globally impactful.
